# Targeting Liver X Receptors for the Treatment of Non-Alcoholic Fatty Liver Disease

**DOI:** 10.3390/cells12091292

**Published:** 2023-05-01

**Authors:** Hyejin Kim, Chaewon Park, Tae Hyun Kim

**Affiliations:** 1 College of Pharmacy, Sookmyung Women’s University, Seoul 04310, Republic of Korea; 2 Drug Information Research Institute, Sookmyung Women’s University, Seoul 04310, Republic of Korea; 3 Muscle Physiome Research Center, Sookmyung Women’s University, Seoul 04310, Republic of Korea

**Keywords:** non-alcoholic fatty liver, liver X receptor, lipid metabolism, inflammation, pharmacological intervention

## Abstract

Non-alcoholic fatty liver disease (NAFLD) refers to a range of conditions in which excess lipids accumulate in the liver, possibly leading to serious hepatic manifestations such as steatohepatitis, fibrosis/cirrhosis and cancer. Despite its increasing prevalence and significant impact on liver disease-associated mortality worldwide, no medication has been approved for the treatment of NAFLD yet. Liver X receptors α/β (LXRα and LXRβ) are lipid-activated nuclear receptors that serve as master regulators of lipid homeostasis and play pivotal roles in controlling various metabolic processes, including lipid metabolism, inflammation and immune response. Of note, NAFLD progression is characterized by increased accumulation of triglycerides and cholesterol, hepatic de novo lipogenesis, mitochondrial dysfunction and augmented inflammation, all of which are highly attributed to dysregulated LXR signaling. Thus, targeting LXRs may provide promising strategies for the treatment of NAFLD. However, emerging evidence has revealed that modulating the activity of LXRs has various metabolic consequences, as the main functions of LXRs can distinctively vary in a cell type-dependent manner. Therefore, understanding how LXRs in the liver integrate various signaling pathways and regulate metabolic homeostasis from a cellular perspective using recent advances in research may provide new insights into therapeutic strategies for NAFLD and associated metabolic diseases.

## 1. Introduction

The liver is a key organ for maintaining systemic energy homeostasis, as it regulates the cellular metabolism of various nutrients including lipids, glucose and proteins [1,2,3]. Hepatic lipid metabolism plays a crucial role in consistently supplying energy sources to other organs by processing food- and adipose tissue-derived lipids and fatty acids (FAs) [1,3]. In the fed state, dietary lipids absorbed from the intestine enter the liver, where they are then metabolized, stored and circulated through the body in the form of triglycerides and cholesterol to provide energy for other peripheral tissues [1,3]. Similarly, under a fasting condition in which dietary energy intake is limited, the liver receives FAs derived from adipose tissue to produce ketone bodies, which are then secreted into circulation and delivered to the brain or heart, where they serve as alternative energy sources to glucose. The net equilibration and homeostasis of the hepatic lipid metabolism is maintained by the dynamic interactions between numerous cellular processes of lipids, including (1) cellular uptake, (2) de novo synthesis of FAs, (3) storage in the forms of triglycerides and/or lipid droplets, (4) mitochondrial FA oxidation and lipolysis and (5) secretion of esterified FAs as triglycerides in the form of very low-density lipoproteins (VLDLs). Disrupting any of these processes can dysregulate the lipid metabolism performed in the liver, leading to the development of metabolic liver disease, such as non-alcoholic fatty liver disease (NAFLD) [2,4,5].

### 1.1. Overview of NAFLD 

Recent studies have revealed that the global prevalence of NAFLD is approximately 25% [4,6,7]. Moreover, as a major cause of chronic liver disease, NAFLD has greatly attributed to an increase in liver disease-associated mortality worldwide, imposing significant health and socioeconomic burdens on patients and society [5,6,8,9,10]. NAFLD refers to a series of liver abnormalities in which excess lipids accumulate within the liver in the absence of excessive alcohol consumption, ranging from non-alcoholic fatty liver (NAFL), non-alcoholic steatohepatitis (NASH), liver fibrosis/cirrhosis and hepatocellular carcinoma (HCC) [4,5,8,11,12]. 

The initial progression of NAFLD arises from the abnormal accumulation of lipids in hepatocytes, a parenchymal cell type of the liver, leading to steatosis (i.e., fatty liver) when the percentage of fat in the tissue increases by more than 5% of total liver weight [4,5,12]. An aberrant increase in intracellular lipids is usually accompanied by an increase in de novo lipogenesis and a decrease in FA oxidation, which results from mitochondrial dysfunction in conjunction with exacerbated oxidative stress. When hepatocytes are chronically exposed to oxidative stress and high levels of lipid peroxidation, they are highly vulnerable to lipotoxic stress-mediated death. In turn, tissue-resident macrophages and infiltrating inflammatory cells are activated upon the release of danger signals from damaged hepatocytes, triggering the secretion of proinflammatory cytokines, which further aggravate hepatocyte damage and death (steatohepatitis). When the liver fails to abrogate accelerated hepatocyte death, hepatic stellate cells are activated in order to compensate for the loss of liver mass by promoting the synthesis and secretion of extracellular matrix molecules such as collagen [4,5,12]. 

With respect to a theoretical hypothesis which may account for the pathogenesis of NAFLD, the “two-hit theory” has been previously considered the most reliable model capable of explaining the onset and progression of NAFLD. In this theory, excessive and sustained accumulation of lipids in hepatocytes (first hit) triggers a lipotoxicity-inflicted inflammatory response, hepatocellular death and fibrogenesis (second hit) [13,14]. However, presently the “multiple-hits theory” is gaining more attention, as it also takes into consideration the contribution of extrahepatic tissues (e.g., the gut-liver-adipose tissue axis) to hepatic manifestation as mediated by several signaling molecules, including gut-derived molecules, cytokines, and adipokines [14]. Interestingly, recent studies have revealed that dysregulation of lipid and/or inflammatory profiles occurs concomitantly during the pathogenesis of NAFLD in various cell types in the liver and other tissues [4,9,11,15], suggesting the importance of intercellular and/or inter-organ crosstalk in the context of lipid and inflammatory signaling pathways in the progression of NAFLD and associated metabolic complications.

Given the significant contribution of the hepatic lipid metabolism and inflammatory signaling to whole-body energy homeostasis, the prevalence of various metabolic complications, including obesity, insulin resistance, type 2 diabetes mellitus (T2DM), hyperlipidemia, hypertension and other cardiovascular diseases, is closely associated with NAFLD. In line with this, it has been well demonstrated that the severity of NAFLD is directly proportional to an increased risk for developing one or more components of metabolic syndrome [10,11,12,16], implying that NAFLD may be a critical driving force of systemic metabolic dysfunction. However, lifestyle modifications—including weight loss, dietary restrictions and exercise—are the only reliable and safe therapeutic options for treating NAFLD. No drugs have been approved for the treatment of NAFLD, owing to limited efficacy and/or safety concerns [17,18,19]. Thus, the identification of novel therapeutic targets and the development of effective and safe pharmacological interventions are urgently needed. 

### 1.2. Nuclear Receptors as Potential Therapeutic Targets for NAFLD 

Among the numerous signaling molecules affected by metabolic stress, nuclear receptors (NRs) have drawn significant attention as potential therapeutic targets for chronic liver disease [20]. NRs belong to a superfamily of ligand-regulated transcription factors; they bind to response elements localized in the cognate promoters of downstream target genes that, in tandem with co-activators and co-repressors, are involved in a variety of intracellular signaling pathways [2,15]. Although several NRs were initially regarded as ‘orphan receptors’ with uncharacterized endogenous ligands, recent advances in molecular biology have enabled us to identify new ligands and gain more insight into the role of novel NRs in the progression of certain diseases.

While NRs are largely categorized into seven subfamilies (NR0-NR6), it is known that some NRs belonging to the NR1 subfamily are implicated in nutrient control and energy homeostasis [14,20,21]. Notably, the majority of NR1 receptors have been identified as lipid-sensing receptors that control downstream target gene expression in response to changes in lipid metabolite levels, such as oxysterols, FA, thyroid hormones, steroid hormones and bile acids [15,20,22,23]. These NR1 receptors include liver X receptor (LXR), peroxisome proliferator-activated receptor (PPAR) and farnesoid X receptor (FXR), and most form a heterodimer with the retinoid X receptor (RXR) upon binding with their cognate ligand [2,15,20].

The NR superfamily has long been regarded as one of the major classes of drug targets for human disease based on its capability of controlling a myriad of biological and pathophysiological processes (e.g., development, metabolism, reproduction, etc.) [14,24] and high feasibility of drug development by replacing small lipophilic molecules with a drug of choice [25]. Notably, numerous studies have demonstrated that disturbances to the hepatic lipid metabolism can be attributed to the dysregulation of lipid metabolism-related signaling pathways driven by lipid-sensing NRs in metabolic diseases, including NAFLD [21,23,26,27,28]. Considering the complicated nature of the etiology of NAFLD pathogenesis, targeting novel NRs that encompass a wide range of cellular signaling pathways related to lipid metabolism in a number of cell types may result in the discovery of promising candidates for developing novel and efficient therapeutic options for NAFLD [2,29,30]. The current review highlights the recent advances made in our understanding of the characteristics and pathophysiological roles of LXRs in the progression of NAFLD from a cellular perspective.

## 2. Liver X Receptor (LXR) as Master Regulator of Whole-Body Metabolism

### 2.1. Overview of LXRs

Cholesterol, a major component of lipids, is important in maintaining physiological homeostasis; the liver plays a crucial role in controlling cellular and systemic cholesterol levels by impacting the systemic metabolism of cholesterol [2,31]. Among the numerous NR1 receptors, LXRs are the most well-known as master physiological regulators of lipid and cholesterol metabolism, which sense and respond to endogenous oxysterol levels [2,14,15,30]. In humans, there are two LXR isoforms—LXRα and LXRβ. In general, LXRα is predominantly expressed in tissues with high metabolic capacity (e.g., the liver, adipose tissue, intestine, kidney, etc.), whereas LXRβ is ubiquitously distributed [2,15,30,32]. However, exact evidence on the direct comparison as to the expression of which isotype of LXRs is more abundant in each metabolic tissue is limited. Emerging evidence from recent studies using LXRα KO and/or LXRβ KO mouse models has revealed that both LXRα and LXRβ usually share their downstream target genes, and they compensate for each other in the transcriptional regulation of certain genes; both LXRs regulate the expression of cholesterol transporters in the same direction, inducing mRNA levels of ABCG5/8 and ABCA1 in the small intestine [33]. Similarly, ABCA1 and SREBP1c gene expression were increased in a parallel manner in either LXRα KO or LXRβ KO mice treated with a pan-LXRα/β agonist [34], suggesting that the identification of specific target genes of either LXRα or LXRβ needs to be further investigated.

LXRα and LXRβ show approximately 77% sequence homology in the DNA-binding domain (DBD) and ligand-binding domain (LBD) [2,15,30,32], indicating high structural similarity. While both LXR isoforms are capable of binding with co-regulators, LXRα interacts more stringently with repressors (e.g., nuclear receptor co-repressor 1 (NCOR1)) [35], whereas LXRβ exerts stronger interactions with the co-activator peptide than with NCOR1 [36]. This notion suggests that the ligand-dependent structural dynamics of the two LXRs are distinct, and the two LXRs may possess different modes of action to release co-repressors and attract co-activators for expressing their target genes [36]. These findings may, at least in part, account for distinctly different phenotypes after selective activation of either LXRα or LXRβ. For example, LXRβ activation increases the gene expression of ABCA1 and SREBP1c in the liver and high-density lipoprotein (HDL) cholesterol levels without significant changes in plasma triglyceride and VLDL content [34]. In line with this, both LXRs are known to inhibit cholesterol uptake in the small intestine by inducing ATP-binding cassette subfamily G number 5/8 (ABCG5/8) and ABCA1 gene expression, suppressing cholesterol uptake into the body. However, selective activation of LXRβ in the small intestine rather increases cholesterol uptake through an incompletely defined mechanism, which seems to be counteracted by LXRα [33]. Collectively, these notions imply that the physiological roles of the two LXRs may be better understood based on their differential tissue expression, which may provide more insight into developing selective agonists of the two LXRs.

Similar to other NR1 receptors, LXRs form a heterodimer with RXRs and bind to an LXR-responsive element (LXRE) in DNA-harboring direct repeats (DRs) of the core sequence AGGTCA spaced by four nucleotides (DR-4) for transcriptional activation [14,15,20,32]. In the absence of ligand binding, the LXR/RXR heterodimer resides within the nucleus, being complexed with co-repressors, such as NCOR1, or silencing mediators of the retinoic acid receptor and thyroid receptor (SMRT, also known as NCOR2), which inhibit transcription by interacting directly with co-repressors or indirectly via histone deacetylase (HDAC) and stress-activated MAPK interacting protein 3A (Sin3A) [15,32,35,37]. Similarly, poly (ADP-ribose) polymerase-1 (PARP-1) was recently identified as another LXR co-repressor; PARP-1-mediated PARylation of LXR affects interactions between the co-activator and co-repressor at downstream target genes such as ABCA1 [36,38]. Upon binding with receptor ligands, the LXR/RXR heterodimer undergoes a conformational change that leads to the dissociation of co-repressors, resulting in the exposure of binding sites to co-activators, such as E1A-associated protein p300 (EP300), and activating signal co-integrator 2 (ASC2; also known as NCOA6) to trigger target gene activation [30,32,35,39]. LXRs are also capable of inhibiting transcription from the promoters of several pro-inflammatory cytokines that do not contain LXREs (i.e., trans-repression) by mediating the recruitment of SUMOylated LXR monomers to target gene promoters [2,32,40,41].

### 2.2. Modulation of LXRs’ Transcriptional Activity

**Figure 1:**
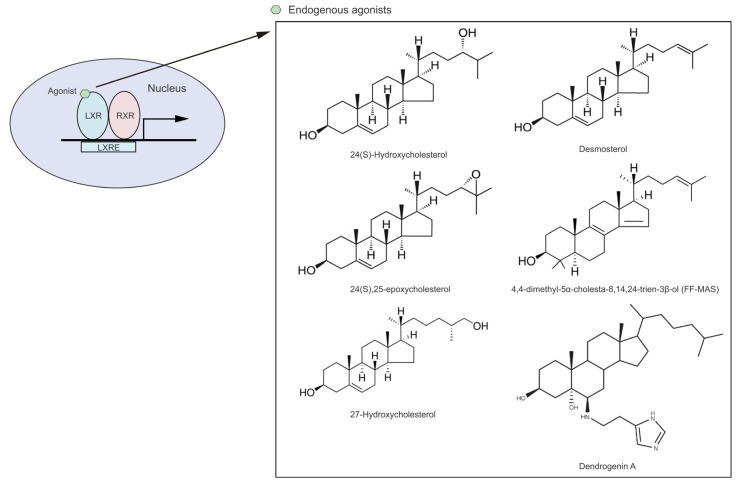
Structures of endogenous agonists for LXRs.

LXRs were initially classified as ‘orphan receptors’ [42,43] until several natural cholesterol derivatives were identified as endogenous agonists [15,20,30]. These cholesterol derivatives are subjected to hydroxylation at either the ring or side chain of their chemical structure to form stronger agonists, including 24(S)-hydroxycholesterol (24-HC, present in the brain and plasma), 24(S),25-epoxycholesterol (present in the liver) and 27(R)-hydroxycholesterol (27-HC, present in macrophages and plasma) [20,30]. Moreover, several intermediate cholesterol metabolites have been newly discovered to be endogenous modulators for LXRs, including desmosterol, 4,4-dimethyl-5α-cholesta-8,14,24-trien-3β-ol (FF-MAS) and dendrogenin A (Figure 1) [44,45]. Notably, while ligand-activated LXRs regulate the expression of genes associated with cholesterol and FA metabolism, highly unsaturated FAs (i.e., polyunsaturated FAs [PUFAs]) inhibit LXR transcriptional activity more effectively than monounsaturated FAs [MUFAs] by competing with oxysterols for receptor binding [46,47]. It has been demonstrated that the essential FA arachidonic acid (20:4, n-6) is known to inhibit LXRα activity, but not LXRβ [47], indicating the varying effect of FAs in modulating LXR activity in accordance with the FA chain length and the degree of saturation.

It has been demonstrated that residues within occupied ligand-binding pockets differ between LXRα and LXRβ, which indicates the high conformational flexibility of the LXR ligand-binding pocket [36]. The plasticity of the ligand-binding pocket of LXRs allows for various synthetic ligands with varying chemical structures to successfully bind, such as T0901317 and GW3965 [30,48,49,50]. Although they have been widely used as synthetic pan-LXR agonists, T0901317 and GW3965 exhibit slightly different efficacies; T0901317 is capable of activating both LXRα and the pregnane X receptor (PXR), while GW3965 serves as selective LXRα/β dual agonist [20,30,51]. Several compounds that modulate LXR activity in a receptor isoform- and/or tissue-specific manner have been identified [30], some of which have been tested or evaluated in numerous clinical trials (Table 1). Notably, because of the multifaceted roles of LXRs in a variety of cellular signaling pathways, both agonists and antagonists of LXRs, such as RGX-104 (agonist), LXR-623 (agonist), SR9238 (inverse agonist), GSK2033 (antagonist) and DUR-928 (larsucosterol, antagonist), exhibit significant regulatory effects on metabolic processes [20,30,52,53,54,55]. While the beneficial effects of LXR agonism are largely attributed to, at least in part, RCT and/or anti-inflammatory functions of LXRs, antagonizing LXRs can be also effective in attenuating hepatic steatosis and consequent fibrosis [20]. For example, DUR-928, a naturally occurring endogenous sulfated oxysterol that antagonizes LXR activity, has been shown to suppress lipogenic genes induction by inhibiting the LXR-SREBP1c axis [56,57,58,59]. Interestingly, several studies have also found that DUR-928 attenuates inflammatory responses by decreasing nuclear factor-κB (NF-κB) nuclear translocation via upregulation of the PPARγ/IκBα signaling pathway [56,57,58,60], alleviating NAFLD. Therefore, further insights into the differential roles of LXRs and their modes of action in each liver cell type are required for the application of LXR modulators in the treatment of NAFLD.

In addition to ligand binding, it has been reported that the transcriptional activity and the stability of LXRs can be controlled via post-translational modifications (PTMs) [30,61]. For instance, deacetylation of LXRs (at K432 in LXRα and K433 in LXRβ) by sirtuin 1 (SIRT1) triggers the ubiquitination-mediated proteasomal degradation of LXRs, which facilitates their removal from target gene promoters, thereby initiating transcription [61,62,63]. Several upstream kinases have been found to phosphorylate LXRs on several Ser/Thr residues; for example, protein kinase A (PKA) induces the phosphorylation of LXRα Ser195/Ser196/Thr290/Ser91, causing receptor degradation and subsequent sterol regulatory element binding protein 1c (*Srebp1c*) gene repression. Moreover, although the exact sites were not identified, LXRα transactivation was regulated in the opposite direction by AMP-activated protein kinase (AMPK) and p70 ribosomal S6 kinase-1 (S6K1) at threonine and serine residues, respectively [64]. The AMPK activator suppressed *Srebp1c* expression and pro-opiomelanocortin (POMC) protein levels mediated by direct phosphorylation of threonine residue(s); [64,65] this regulation was abrogated by S6K1, which induces phosphorylation of serine residues and consequently increases transactivation of downstream target genes of LXRα [64]. In addition, physiological concentrations of glucose activate LXRs in the liver through O-GlcNAcylation, which induces the expression of genes involved in lipid and cholesterol metabolism [66,67]. O-GlcNAc moieties are attached to serine/threonine residues by O-GlcNAc transferase (OGT) [68], indicating that O-GlcNAcylation and phosphorylation may act in tandem to control LXR activity. LXRs can inhibit pro-inflammatory gene expression (termed ‘trans-repression’) via SUMOylation [30,69]. SUMOylation is promoted by binding endogenous LXR ligands, including 22(R)-hydroxycholesterol, 24(S),25-epoxycholesterol and 24(S)-hydroxycholesterol, leading to interaction with the NCOR co-repressor [30,69].

### 2.3. Regulation of Cellular Processes by LXRs: An Overview

Decades of studies have revealed that LXRs serve as master regulators of multiple signaling pathways, such as lipid metabolism, inflammation and immunity, which shows the multifaceted functionality of LXRs in the regulation of overall metabolic processes. In particular, hepatic lipid content is directly proportional to the severity of a variety of metabolic diseases in the context of overnutrition [70,71]. Likewise, it is noted that LXR expression positively correlates with the degree of fat disposition, inflammation and fibrosis in the liver of NAFLD patients [72], suggesting a significant impact on liver pathophysiology. Furthermore, given the pivotal contribution of NAFLD progression in systemic metabolic disorders, LXRs may play crucial roles in human health and disease by modulating not only the hepatic lipid metabolism, but also numerous metabolic signaling pathways.

#### 2.3.1. Roles of LXRs in FA Metabolism

At the cellular level, the net amount of fat is largely determined by the complex interplay between a variety of cellular processes associated with lipid metabolism, including de novo lipogenesis, FA oxidation, lipolysis and the import/export of lipid species [73]. The onset of NAFLD generally begins with abnormal fat accumulation in hepatocytes, where a large amount of FFAs derived from either excess dietary lipids or increased lipolysis in adipose tissue enter via fatty acid translocase (FAT/CD36) [74]. In addition, it is also reported that CD36 is a shared downstream target of LXRα, PXR and PPARγ [74], and that LXRα-mediated CD36 induction is responsible for T0901317 treatment-mediated hepatic steatosis, which was abrogated in CD36 KO mice [74].

LXRs have been established to play an essential role in controlling lipogenic genes in the liver [75,76,77,78], as supported by the observation that administrating T0901317 (a synthetic pan agonist of LXRs) to mice increased triglyceride levels in both liver tissue and plasma through enhanced FA biosynthesis [76,78]. The stimulation of lipogenesis by LXRs largely occurs through the transcriptional induction of lipogenic target genes, such as SREBP1c, fatty acid synthase (FASN), acetyl-CoA carboxylase (ACC) and stearoyl-CoA desaturase-1 (SCD1) [15,76,77,79]. Interestingly, the transcriptional activity of most lipogenic genes is induced via the LXR-SREBP1c axis; however, LXRs can also increase target gene expression by directly binding to LXRE sites in their promoter regions. A recent study showed that hepatic de novo lipogenesis was significantly upregulated in obese patients with NAFLD compared to obese individuals without NAFLD, whereas the proportion of hepatic triglyceride synthesis from either dietary fat or plasma free fatty acids (FFAs) was comparable [80,81]. In mice, LXR agonist-mediated increases in hepatic triglyceride levels were not fully ameliorated by the co-administration of a PPARα ligand [82], implicating the significant contribution of lipogenesis in the development of fatty liver disease.

Recent studies have found that LXRs modulate FA catabolism in various tissues via distinct mechanisms. LXRα-knockout (KO) mice fed a high-fat and high-cholesterol diet (HFHC) showed increased energy expenditure and UCP expression in muscle and adipose tissue [78,83]. Furthermore, in vivo results from wild-type mice subjected to long-term treatment of GW3965 demonstrated the inhibitory effect of LXRs on energy expenditure and UCP1 expression in brown adipose tissue, which was also confirmed in LXRα/β double KO mice fed a high-carbohydrate diet [84]. More recently, LXRα activation in hepatocytes was shown to suppress mitochondrial β-oxidation by inhibiting autophagy and lipophagy, leading to excessive fat accumulation in the liver [85]. On the contrary, it has been also reported that LXRα activation indirectly facilitates mitochondrial β-oxidation and lipolysis in white adipose tissue by transcriptionally inducing pyruvate dehydrogenase kinase 4 (PDK4) expression, which in turn switches from pyruvate dehydrogenase complex-dependent glucose oxidation to lipid oxidation [86]. Collectively, LXRs contribute to the regulation of lipid homeostasis in various metabolic tissues by balancing the anabolism and catabolism of FAs.

#### 2.3.2. Roles of LXRs in Cholesterol Metabolism

Initial studies delineating the regulatory role of oxysterol as an LXR physiological ligand have demonstrated its possible involvement in maintaining cholesterol homeostasis [32,87,88]. A marked increase in cholesteryl ester levels in the livers of LXRα-KO mice fed a high-cholesterol diet further confirmed the significant contribution of LXRα to cholesterol metabolism [79]. The excessive accumulation of hepatic cholesterol due to the loss of LXRα, but not LXRβ, was attributed to a lack of cytochrome P450 7A1 (CYP7A1) expression, the rate-limiting enzyme responsible for the conversion of cholesterol to bile acid [32,78,89]. This step is critical for clearing cholesterol from the body, as it cannot be catabolized by animal cells [15]. Subsequently, bile acid, which is synthesized from cholesterol, is subjected to biliary excretion via ABCG5 and ABCG8 [15,90]. LXRs induce ABCG5/G8 expression in both the canalicular membrane of hepatocytes and the apical membrane of enterocytes, facilitating biliary excretion and inhibiting the intestinal absorption of cholesterol, respectively [15,30,32,91]. Similarly, loss of either LXRs or ABCG5/8 impairs biliary sterol trafficking and fecal sterol excretion [90,92,93], further demonstrating the important role of LXRs in cholesterol absorption and excretion in vivo.

**Figure 2:**
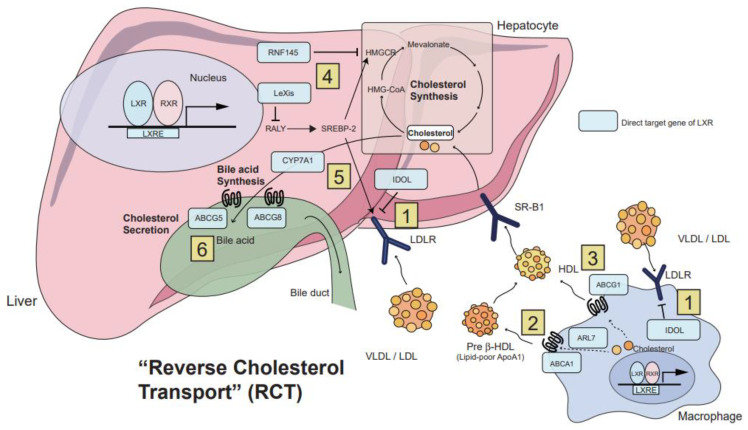
The control of cholesterol uptake, transport and excretion by LXRs. The fine-tuned regulation of cholesterol levels in the body is a consequence of integrated interaction among various cell types and tissues, such as macrophage, liver and intestine, and LXRs play a pivotal role in these processes, termed as reverse cholesterol transport (RCT). Some, but not all, target genes that are directly regulated by LXRs are highlighted in rectangles colored in light blue. (1) LXR agonism inhibits cholesterol uptake in the liver and macrophages via upregulating the inducible degrader of the LDL receptor (IDOL), which degrades the low-density lipoprotein receptor (LDLR). (2) In parallel, LXR activation also promotes cholesterol efflux from macrophages by inducing ATP-binding cassette subfamily A member 1 (ABCA1) and ADP-ribosylation factor-like protein 7 (ARL7), facilitating cholesterol transport to lipid-poor apolipoprotein A1 (ApoA1) or pre-β high density lipoprotein (pre-β HDL). (3) Similarly, ABCG1, another direct target of LXR, also promotes cholesterol transport to ApoA1-containing lipoprotein to form mature HDL. (4) In line with this, LXR activation in the liver suppresses cholesterol biosynthesis by transcriptional induction of E3 ubiquitin protein ligase RING finger protein 145 (RNF145) and liver-expressed, LXR-induced sequence (Lexis). (5) Moreover, hepatic LXR induces cytochrome P450 7A1 (CYP7A1) expression, which promotes cholesterol clearance via conversion of cholesterol into bile acids. (6) Bile acids derived from cholesterol are then subjected to biliary excretion through ABCG5 and ABCG8, both of which are another direct target of LXR.

In order to excrete excess cholesterol out of the body, surplus cholesterol in peripheral tissues needs to be transferred to the liver for ultimate clearance through biliary excretion, the overall process of which is called ‘reverse cholesterol transport (RCT)’ [32,78]. LXRs participate in the initial steps of RCT through upregulating the expression of a cluster of transporter genes, such as ABCA1 and ABCG1, favoring the transfer of free cholesterol to pre-HDL [78,94,95]. ABCA1 and ABCG1 are direct LXR target genes that work together in lipid-laden macrophages on the efflux of cholesterol to apolipoprotein A1 (ApoA1) and HDL, respectively [2,32,96,97]. A lipid-poor ApoA1 becomes nascent HDL when being complexed with cholesterol derived from macrophages through ABCA1, which can further develop into mature HDL after being loaded with cholesterol exported from macrophages via ABCG1 [31,78,94]. HDL cholesterol is then taken up by the liver through direct binding to the scavenger receptor class B type 1 (SR-B1) that selectively removes cholesteryl ester from HDL [2,32]. LXRs also regulate ADP-ribosylation factor-like protein 7 (ARL7), which promotes the transport of excess cholesterol across the membrane through ABCA1 [2,98]. Additionally, LXRs are capable of inducing the expression of a subset of genes associated with apolipoproteins (i.e., ApoE and ApoC1) and lipoprotein remodeling (e.g., cholesteryl ester transfer protein [CETP] and lipoprotein lipase [LPL]) [15,91,99,100], suggesting that the integrated regulation of these genes by LXR activation facilitates RCT from the cell periphery to the liver (Figure 2).

Following the RCT procedure, cholesterol bound to low-density lipoprotein (LDL) is transported to the liver, where LDL receptors (LDLR) are expressed, indicating that the regulation of LDLR expression is also essential for cholesterol uptake. LXRs participate in the regulation of cholesterol uptake by upregulating the expression of inducible degrader of LDLR (IDOL), an E3 ubiquitin protein ligase responsible for the degradation of LDLR and the VLDL receptor (VLDLR) [101,102,103]. Of note, the modulation of IDOL expression by LXR activation shows differential responses in a species-dependent manner; decreased hepatic LDLR expression in conjunction with increased plasma cholesterol levels was observed by LXR activation in primates and humans, while such regulation was not confirmed in rodents [104,105]. Additional investigation is required to determine whether the LXR-IDOL pathway could be a promising therapeutic target for modulating plasma lipid levels in humans.

In addition to cholesterol transport and elimination, recent studies have demonstrated that LXRs can modulate cholesterol biosynthesis in concert with SREBP2 in response to changes in cholesterol levels [78]. LXRs and SREBP2 work dynamically together to maintain cellular levels of cholesterol. SREBP2 triggers the uptake and synthesis of cholesterol when cholesterol levels are low and positively regulates ABCA1 gene transcription by producing oxysterol ligands of LXRs [78,106]. In contrast, LXRs suppress cholesterol biosynthesis by inhibiting the protein maturation of SREBP2 and its transport to the Golgi apparatus via induction of the RING finger protein 145 (RNF145), an E3 ubiquitin protein ligase [107]. In parallel, LXRs also increase non-coding RNA LXR-induced sequences (Lexis), resulting in the inhibition of the transcriptional activity of genes involved in cholesterol biosynthesis [108]. Collectively, LXRs play a crucial role in managing cholesterol at both cellular and systemic levels.

#### 2.3.3. Roles of LXRs in Inflammatory and Immune Responses

In addition to their role in the management of lipid metabolism, LXRs have been shown to inhibit a set of pro-inflammatory signaling pathways upon bacterial infection or exposure to numerous cytokines in macrophages [30,32,109]. Both isoforms of LXR exert anti-inflammatory properties, as supported by research showing the inhibition of a subset of pro-inflammatory genes (e.g., inducible nitric oxide synthase (iNOS), metalloproteinase-9 (MMP-9) and cyclooxygenase-2 [COX-2]) by LXR ligands in macrophages derived from wild-type, LXRα-KO and LXRβ-KO mice, but not from LXRα/β double-KO mice [30,32,109,110,111]. The anti-inflammatory activity of LXRs is largely mediated by the transcriptional suppression of NF-κB, the key transcription factor that governs proinflammatory signaling pathways; however, LXREs have not been found in the promoter-proximal regions of these proinflammatory genes [30,32]. Emerging evidence has further indicated that LXRs primarily exert anti-inflammatory activity in several different cell types, including the liver, where acute phase response protein expression is inhibited by LXRs [30,112,113]. As such, long-term activation of LXRs suppress graft-versus-host inflammatory reactions, chronic damage and immune responses in kidney allografts in rodents [30,114]. It has been demonstrated that reciprocal regulation exists between Toll-like receptor 3/4 (TLR3/4) signaling by microbial ligands and LXR-dependent cholesterol metabolism [32,115]. LXR activation has been found to blunt the TLR-dependent inflammatory response by inducing ABCA1, whereas activation of TLR3/4 during bacterial or viral infection of macrophages significantly suppressed LXR and downstream target gene expression via induction of interferon regulatory factor 3 (IRF3) [32,116,117]. Cumulative evidence has also found that LXRs play an important role in the pathogen type-dependent immune response [30,32]. Notably, LXRα exhibits a protective function in macrophages against bacterial or viral infection by inducing the expression of anti-apoptotic factors (e.g., Spα), and numerous studies have shown that LXRα-KO macrophages are more vulnerable to bacterial infection-mediated inflammatory responses and/or cell death [118,119,120,121,122,123]. As such, these studies together highlight the crucial regulatory functions of LXRs at the crossroads of metabolism, inflammation and immunity.

## 3. LXRs in the Progression of NAFLD

Emerging studies have demonstrated how NAFLD is a major hepatic manifestation of various metabolic disorders, some of which constitute metabolic syndrome [20]. It is well known that the progression from a healthy liver to NAFL, NASH and fibrosis/cirrhosis develops as a consequence of integrated metabolic alterations that occur concurrently and dynamically in a variety of liver and other tissue cell types. In particular, a growing body of evidence has revealed that hepatic LXRs hold a critical position in NAFLD progression, based on the significant roles of LXRs in lipid metabolism and inflammatory signaling. According to a previous study, hepatic LXR expression is highly correlated with disease severity in patients with NAFLD, as assessed by hepatic lipid content, hepatic inflammation and fibrosis [72]. However, this finding may need to be carefully interpreted, as the observed LXR expression noted in the study is the integrated gene expression of multiple liver tissue cell types, which exhibit common but differential functions of LXR. It is noteworthy that, as previously mentioned above, the anti-inflammatory function of LXRs may not entirely account for the positive correlation between hepatic LXR expression and the severity of clinical manifestations of NAFLD (i.e., hepatic steatosis, inflammation and fibrosis). This seemingly contradictory notion can be partially explained by some recent evidence; while the anti-inflammatory role of LXRs is primarily executed in macrophages and immune cells, inflammatory signaling is capable of promoting LXRα transcriptional activity in hepatocytes through c-Jun N-terminal protein kinase 1 (JNK1)-dependent LXRα phosphorylation, inducing fat accumulation in the liver [124]. In line with this, this triglyceride accumulation is known to protect cells against FA-induced lipotoxicity [125], suggesting that hepatic LXR expression can be upregulated as an adaptive response following lipotoxic and inflammatory stimuli in the course of NAFLD progression. Taken together, the diverse and complicated functions of LXRs and their expression profile need to be understood in a cell type- and pathological condition-dependent manner.

Although the increased accumulation of cholesterol and triglycerides in the liver is frequently observed in the setting of NAFLD [2], the overall signaling pathways that regulate either cholesterol or FA levels by LXRs show highly divergent and complex patterns depending on the primary function of LXRs in each cell type. For instance, when LXR signaling is impaired, the pool of cholesterol accumulated within macrophages might not be properly excreted due to diminished expression of ABCA1/ABCG1, triggering the development of lipid-laden macrophages (i.e., foam cell) and the detrimental inflammatory response [30,31,32]. Similarly, defects in LXR signaling in hepatocytes are also attributed to increased accumulation of cholesterol due to decreased CYP7A1 activity, promoting hepatocellular injury and the secretion of chemokines and inflammatory cytokines [30,31,32]. Instead, the impaired LXRs-SREBP1c axis in hepatocytes inhibits FA synthesis which ameliorates hepatic steatosis, indicating that LXRs can differentially control cholesterol and FA metabolism in the context of NAFLD progression.

Several studies have recently suggested the role of LXRs as a gatekeeper in the progression from steatosis to steatohepatitis/fibrosis. While increased accumulation of free cholesterol in hepatic stellate cells induces trans-differentiation into myofibrogenic phenotypes, which exacerbates liver fibrosis [126,127], LXRβ activation in hepatic stellate cells largely exerts antifibrogenic and anti-inflammatory properties [128], possibly preventing the progression to liver fibrosis. Similarly, LXR activation in hepatocytes suppressed the transactivation of a subset of NASH-promoting genes by destabilizing the TAZ (WWTR1) protein level, halting the progression from steatosis to steatohepatitis [129]. Likewise, when mice carrying a phosphorylation mutant of LXRα at the Ser196 residue (Ser198 in humans) were subjected to HFHC diet feeding, the progression to hepatic inflammation and fibrosis was abrogated while hepatic non-esterified fatty acids (NEFA) and triglycerides were significantly elevated [130]. Collectively, these findings imply that LXRs pose an attractive therapeutic target for NAFLD, possibly enabling the reversal of progression toward more severe forms of disease.

### Considerations of Targeting LXRs for the Treatment of NAFLD

Given their multifaceted activities in numerous cellular processes, LXRs are regarded as promising therapeutic targets for various human diseases, including NAFLD. Oxysterols are oxidized cholesterol derivatives that are generated during the early stages of cholesterol metabolism and bile acid synthesis [131], and as already described above, they exert numerous metabolic functions through binding to numerous NR1 receptors, including LXRs [20,22,23]. Emerging evidence has shown that hepatic and/or serum levels of several oxysterols are elevated in patients with NAFLD, and some of them are known to serve as LXR modulators, including 4β-hydroxycholesterol (4β-HC), 25-hydroxycholesterol (25-HC) and 27-HC [131,132]. Although it has long been well established that oxysterols play a key role in various metabolic processes (e.g., lipid metabolism, RCT, cholesterol metabolism and others), their precise roles in NAFLD pathogenesis remains unclear [131]. For example, 4β-HC has been shown to promote lipogenic gene induction in an SREBP1c-dependent manner in hepatocytes [133], while 27-HC inhibits SREBP1c activation and concomitant suppression of lipogenic genes, alleviating hepatic lipid accumulation in mice [134]. More interestingly, the effect of 25-HC on cellular lipid accumulation has shown contradictory outcomes via different mechanisms; 25-HC was found to inhibit SREBP maturation through direct interaction with the SREBP cleavage-activating protein (SCAP) [135], while other studies reported that 25-HC serves as a ligand for DNA methyltransferase-1 (DNMT1), mediating high glucose-induced lipid accumulation [56,136]. Overall, these findings suggest that further investigations need to be conducted to elucidate the exact role of oxysterols in the pathogenesis of NAFLD.

In line with this, owing to LXRs’ divergent functions, synthetic modulators of LXRs can cause undesired outcomes or ones that might interfere with other intended therapeutic efficacies. For example, although first-generation synthetic agonists of LXRs showed benefits in cholesterol clearance (i.e., RCT), hepatic de novo lipogenesis and plasma triglyceride levels were also significantly increased [78,137]. Of note, T0901317 treatment in LXRα KO mice showed marked anti-atherogenic efficacy as opposed to a mild effect on plasma triglyceride levels, suggesting the potential of LXRβ activation in dissecting anti-atherogenic and lipogenic action in response to treatment with a pan-LXR agonist.

In spite of the high degree of sequence homology in the ligand-binding domain between the two LXRs, many studies which develop selective LXRβ agonists have been performed [2,138,139,140]. As briefly mentioned above, LXRβ is ubiquitously distributed throughout the body, which makes it difficult to selectively modulate LXRβ activity rather than LXRα under comparable pharmacokinetic profiles. Thus, another hypothesis has been proposed to bypass undesirable effects of LXR agonists with regard to increases in hepatic lipogenesis. As part of these efforts, several selective LXR agonists have been developed and examined. For example, based on the knowledge that the RCT procedure is facilitated by multiple tissues and cell types (e.g., macrophage, liver and intestine), GW6340, an intestine-selective LXR agonist, has shown an enhanced efficacy on cholesterol efflux by inducing intestinal ABCG5/ABCG8, with no such changes in hepatic lipogenesis in mice [92]. Likewise, other pharmaceutical interventions such as desmosterol, LXR-623, CS-8080 and BMS-779788 have also been examined, although they have been used less frequently in the treatment of NAFLD and some of these agents have been dropped from clinical trials owing to unexpected adverse results such as neurological effects (e.g., confusion, drowsiness, diminished comprehension, etc.), elevated plasma and hepatic lipids, decreased circulating neutrophils and other undisclosed safety concerns [2,78,104,141,142,143,144,145,146]. Moreover, recent studies have identified potential benefits of targeting LXRs in combination with other reagents, including glucocorticoids [147]. Thus, further optimization of the development of tissue-selective LXR agonists may provide a better therapeutic option to acquire potent, safe and specific efficacy without any unintended side effects.

## 4. Conclusions

Given the complicated etiology of NAFLD progression, the identification of novel targets, the activity of which potentially shows significant correlation with respect to the severity of NAFLD, will be of great value in developing therapeutic strategies to overcome metabolic disorders. Emerging evidence from recent preclinical and clinical studies has now demonstrated the crucial role of LXRs in a variety of cellular processes involved in maintaining metabolic homeostasis. However, our understanding of the comprehensive role of LXRs in the context of cell type-specific and cell-to-cell communication is still incomplete, and there are some setbacks and limitations in the current pharmacological intervention strategy. A tissue- and/or isoform-selective modulation of LXR pathways with minimized side effects may pose a promising potential to achieve beneficial clinical outcomes, which will be of great value in developing therapeutic strategies against NAFLD and associated metabolic complications.

## Figures and Tables

**Table 1:** Ongoing clinical trials of pharmacotherapies by targeting LXRs.

Primary Mechanism	Agent (Trial Name)	Structure	Clinical Trials	NCT Number (ClinicalTrilas.gov)	
Partial LXRα and full LXRβ agonist	LXR-623	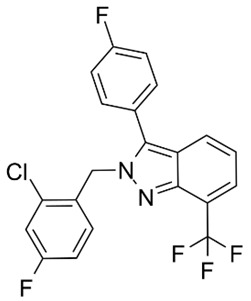	Phase 1 in healthy adults	NCT00366522	https://clinicaltrials.gov/ct2/results?cond=NCT00366522&term=&cntry=&state=&city=&dist= (accessed on 30 March 2023)
			Phase 1 in healthy subjects	NCT00379860	https://clinicaltrials.gov/ct2/results?cond=NCT00379860&term=&cntry=&state=&city=&dist= (accessed on 30 March 2023)
			Phase 1 in healthy Japanese males	NCT00385489	https://clinicaltrials.gov/ct2/results?cond=NCT00385489&term=&cntry=&state=&city=&dist= (accessed on 30 March 2023)
Partial LXRα and LXRβ agonist	CS-8080	Not known	Phase 1 in healthy adults	NCT00613431	https://clinicaltrials.gov/ct2/results?cond=NCT00613431&term=&cntry=&state=&city=&dist= (accessed on 30 March 2023)
			Phase 1 in healthy subjects	NCT00796575	https://clinicaltrials.gov/ct2/results?cond=NCT00796575&term=&cntry=&state=&city=&dist= (accessed on 30 March 2023)
LXRα-selective agonist	BMS-779788	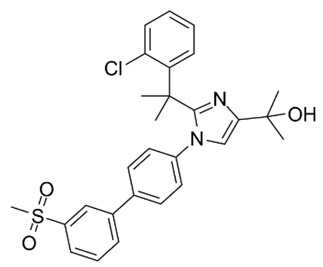	Phase 1 in patients with atherosclerosis	NCT00836602	https://clinicaltrials.gov/ct2/results?cond=NCT00836602&term=&cntry=&state=&city=&dist= (accessed on 30 March 2023)
LXRβ-selective agonist	BMS-852927	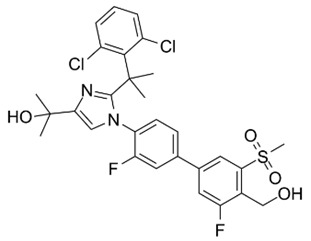	Phase 1 in patients with hypercholesterolemia	NCT01651273	https://clinicaltrials.gov/ct2/results?cond=NCT01651273&term=&cntry=&state=&city=&dist= (accessed on 30 March 2023)
Potent LXR antagonist	DUR-928	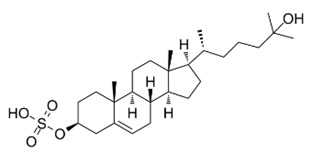	Phase 2 in patients with alcoholic steatosis	NCT03917407	https://clinicaltrials.gov/ct2/results?cond=NCT03917407&term=&cntry=&state=&city=&dist= (accessed on 30 March 2023)
			Phase 2 in patients with alcoholic steatosis	NCT04563026	https://clinicaltrials.gov/ct2/results?cond=NCT04563026&term=&cntry=&state=&city=&dist= (accessed on 30 March 2023)
			Phase 2 in patients with alcoholic steatosis	NCT03432260	https://clinicaltrials.gov/ct2/results?cond=NCT03432260&term=&cntry=&state=&city=&dist= (accessed on 30 March 2023)

## Data Availability

Data sharing not applicable.

## References

[1] Rui L. (2014). Energy metabolism in the liver. Compr. Physiol..

[2] Hong C., Tontonoz P. (2014). Liver X receptors in lipid metabolism: Opportunities for drug discovery. Nat. Rev. Drug Discov..

[3] Bechmann L.P., Hannivoort R.A., Gerken G., Hotamisligil G.S., Trauner M., Canbay A. (2012). The interaction of hepatic lipid and glucose metabolism in liver diseases. J. Hepatol..

[4] Loomba R., Friedman S.L., Shulman G.I. (2021). Mechanisms and disease consequences of nonalcoholic fatty liver disease. Cell.

[5] Parlati L., Régnier M., Guillou H., Postic C. (2021). New targets for NAFLD. JHEP Rep..

[6] Younossi Z., Anstee Q.M., Marietti M., Hardy T., Henry L., Eslam M., George J., Bugianesi E. (2018). Global burden of NAFLD and NASH: Trends, predictions, risk factors and prevention. Nat. Rev. Gastroenterol. Hepatol..

[7] De Jesus D.F., Orime K., Kaminska D., Kimura T., Basile G., Wang C., Haertle L., Riemens R., Brown N.K., Hu J. (2020). Parental metabolic syndrome epigenetically reprograms offspring hepatic lipid metabolism in mice. J. Clin. Investig..

[8] Younossi Z.M. (2019). Non-alcoholic fatty liver disease—A global public health perspective. J. Hepatol..

[9] Francque S., Szabo G., Abdelmalek M.F., Byrne C.D., Cusi K., Dufour J., Roden M., Sacks F., Tacke F. (2021). Nonalcoholic steatohepatitis: The role of peroxisome proliferator-activated receptors. Nat. Rev. Gastroenterol. Hepatol..

[10] Adams L.A., Anstee Q.M., Tilg H., Targher G. (2017). Non-alcoholic fatty liver disease and its relationship with cardiovascular disease and other extrahepatic diseases. Gut.

[11] Marchesini G., Bugianesi E., Forlani G., Cerrelli F., Lenzi M., Manini R., Natale S., Vanni E., Villanova N., Melchionda N. (2003). Nonalcoholic fatty liver, steatohepatitis, and the metabolic syndrome. Hepatology.

[12] Angulo P. (2002). Nonalcoholic fatty liver disease. N. Engl. J. Med..

[13] Day C.P., James O.F. (1998). Steatohepatitis: A tale of two “hits”?. Gastroenterology.

[14] Cariello M., Piccinin E., Moschetta A. (2021). Transcriptional Regulation of Metabolic Pathways via Lipid-Sensing Nuclear Receptors PPARs, FXR, and LXR in NASH. Cell Mol. Gastroenterol. Hepatol..

[15] Calkin A.C., Tontonoz P. (2012). Transcriptional integration of metabolism by the nuclear sterol-activated receptors LXR and FXR. Nat. Rev. Mol. Cell Biol..

[16] Ratziu V., Bellentani S., Cortez-Pinto H., Day C., Marchesini G. (2010). A position statement on NAFLD/NASH based on the EASL 2009 special conference. J. Hepatol..

[17] Sanyal A.J., Chalasani N., Kowdley K.V., McCullough A., Diehl A.M., Bass N.M., Neuschwander-Tetri B.A., Lavine J.E., Tonascia J., Unalp A. (2010). Pioglitazone, vitamin E, or placebo for nonalcoholic steatohepatitis. N. Engl. J. Med..

[18] Neuschwander-Tetri B.A., Loomba R., Sanyal A.J., Lavine J.E., Van Natta M.L., Abdelmalek M.F., Chalasani N., Dasarathy S., Diehl A.M., Hameed B. (2015). Farnesoid X nuclear receptor ligand obeticholic acid for non-cirrhotic, non-alcoholic steatohepatitis (FLINT): A multicentre, randomised, placebo-controlled trial. Lancet.

[19] Oseini A.M., Sanyal A.J. (2017). Therapies in non-alcoholic steatohepatitis (NASH). Liver Int..

[20] Tanaka N., Aoyama T., Kimura S., Gonzalez F.J. (2017). Targeting nuclear receptors for the treatment of fatty liver disease. Pharmacol. Ther..

[21] Sugden M.C., Holness M.J. (2008). Role of nuclear receptors in the modulation of insulin secretion in lipid-induced insulin resistance. Biochem. Soc. Trans..

[22] Evans R.M., Mangelsdorf D.J. (2014). Nuclear Receptors, RXR, and the Big Bang. Cell.

[23] Beaven S.W., Tontonoz P. (2006). Nuclear receptors in lipid metabolism: Targeting the heart of dyslipidemia. Annu. Rev. Med..

[24] Mangelsdorf D.J., Thummel C., Beato M., Herrlich P., Schütz G., Umesono K., Blumberg B., Kastner P., Mark M., Chambon P. (1995). The nuclear receptor superfamily: The second decade. Cell.

[25] Sladek F.M. (2003). Nuclear receptors as drug targets: New developments in coregulators, orphan receptors and major therapeutic areas. Expert Opin. Ther. Targets.

[26] Aranda A., Pascual A. (2001). Nuclear hormone receptors and gene expression. Physiol. Rev..

[27] Portincasa P., Grattagliano I., Palmieri V.O., Palasciano G. (2006). Current pharmacological treatment of nonalcoholic fatty liver. Curr. Med. Chem..

[28] Hong T., Chen Y., Li X., Lu Y. (2021). The Role and Mechanism of Oxidative Stress and Nuclear Receptors in the Development of NAFLD. Oxidative Med. Cell. Longev..

[29] Gronemeyer H., Gustafsson J., Laudet V. (2004). Principles for modulation of the nuclear receptor superfamily. Nat. Rev. Drug Discov..

[30] Jakobsson T., Treuter E., Gustafsson J., Steffensen K.R. (2012). Liver X receptor biology and pharmacology: New pathways, challenges and opportunities. Trends Pharmacol. Sci..

[31] Luo J., Yang H., Song B. (2020). Mechanisms and regulation of cholesterol homeostasis. Nat. Rev. Mol. Cell Biol..

[32] Zelcer N., Tontonoz P. (2006). Liver X receptors as integrators of metabolic and inflammatory signaling. J. Clin. Investig..

[33] Hu X., Steffensen K.R., Jiang Z.-Y., Parini P., Gustafsson J.-Å., Gåfvels M., Eggertsen G. (2012). LXRβ activation increases intestinal cholesterol absorption, leading to an atherogenic lipoprotein profile. J. Intern. Med..

[34] Quinet E.M., Savio D.A., Halpern A.R., Chen L., Schuster G.U., Gustafsson J., Basso M.D., Nambi P. (2006). Liver X receptor (LXR)-beta regulation in LXRalpha-deficient mice: Implications for therapeutic targeting. Mol. Pharmacol..

[35] Hu X., Li S., Wu J., Xia C., Lala D.S. (2003). Liver X receptors interact with corepressors to regulate gene expression. Mol. Endocrinol..

[36] Buñay J., Fouache A., Trousson A., de Joussineau C., Bouchareb E., Zhu Z., Kocer A., Morel L., Baron S., Lobaccaro J.A. (2021). Screening for liver X receptor modulators: Where are we and for what use?. Br. J. Pharmacol..

[37] Wen Y.D., Perissi V., Staszewski L.M., Yang W.M., Krones A., Glass C.K., Rosenfeld M.G., Seto E. (2000). The histone deacetylase-3 complex contains nuclear receptor corepressors. Proc. Natl. Acad. Sci. USA.

[38] Shrestha E., Hussein M.A., Savas J.N., Ouimet M., Barrett T.J., Leone S., Yates J.R., Moore K.J., Fisher E.A., Garabedian M.J. (2016). Poly(ADP-ribose) Polymerase 1 Represses Liver X Receptor-mediated ABCA1 Expression and Cholesterol Efflux in Macrophages. J. Biol. Chem..

[39] Lee S., Lee J., Lee S., Lee J.W. (2008). Activating signal cointegrator-2 is an essential adaptor to recruit histone H3 lysine 4 methyltransferases MLL3 and MLL4 to the liver X receptors. Mol. Endocrinol..

[40] Kidani Y., Bensinger S.J. (2012). Liver X receptor and peroxisome proliferator-activated receptor as integrators of lipid homeostasis and immunity. Immunol. Rev..

[41] Gabbi C., Warner M., Gustafsson J. (2014). Action mechanisms of Liver X Receptors. Biochem. Biophys. Res. Commun..

[42] Apfel R., Benbrook D., Lernhardt E., Ortiz M.A., Salbert G., Pfahl M. (1994). A novel orphan receptor specific for a subset of thyroid hormone-responsive elements and its interaction with the retinoid/thyroid hormone receptor subfamily. Mol. Cell Biol..

[43] Teboul M., Enmark E., Li Q., Wikström A.C., Pelto-Huikko M., Gustafsson J.A. (1995). OR-1, a member of the nuclear receptor superfamily that interacts with the 9-cis-retinoic acid receptor. Proc. Natl. Acad. Sci. USA.

[44] Segala G., David M., de Medina P., Poirot M.C., Serhan N., Vergez F., Mougel A., Saland E., Carayon K., Leignadier J. (2017). Dendrogenin A drives LXR to… trigger leth.hal autophagy in cancers. Nat. Commun..

[45] Yang C., McDonald J.G., Patel A., Zhang Y., Umetani M., Xu F., Westover E.J., Covey D.F., Mangelsdorf D.J., Cohen J.C. (2006). Sterol intermediates from cholesterol biosynthetic pathway as liver X receptor ligands. J. Biol. Chem..

[46] Bedi S., Hines G.V., Lozada-Fernandez V.V., de Jesus Piva C., Kaliappan A., Rider S.D., Hostetler H.A. (2017). Fatty acid binding profile of the liver X receptor α. J. Lipid Res..

[47] Pawar A., Xu J., Jerks E., Mangelsdorf D.J., Jump D.B. (2002). Fatty acid regulation of liver X receptors (LXR) and peroxisome proliferator-activated receptor alpha (PPARalpha) in HEK293 cells. J. Biol. Chem..

[48] Williams S., Bledsoe R.K., Collins J.L., Boggs S., Lambert M.H., Miller A.B., Moore J., McKee D.D., Moore L., Nichols J. (2003). X-ray crystal structure of the liver X receptor beta ligand binding domain: Regulation by a histidine-tryptophan switch. J. Biol. Chem..

[49] Svensson S., Ostberg T., Jacobsson M., Norström C., Stefansson K., Hallén D., Johansson I.C., Zachrisson K., Ogg D., Jendeberg L. (2003). Crystal structure of the heterodimeric complex of LXRalpha and RXRbeta ligand-binding domains in a fully agonistic conformation. EMBO J..

[50] Färnegårdh M., Bonn T., Sun S., Ljunggren J., Ahola H., Wilhelmsson A., Gustafsson J., Carlquist M. (2003). The three-dimensional structure of the liver X receptor beta reveals a flexible ligand-binding pocket that can accommodate fundamentally different ligands. J Biol. Chem..

[51] Shenoy S.D., Spencer T.A., Mercer-Haines N.A., Alipour M., Gargano M.D., Runge-Morris M., Kocarek T.A. (2004). CYP3A induction by liver x receptor ligands in primary cultured rat and mouse hepatocytes is mediated by the pregnane X receptor. Drug Metab. Dispos..

[52] Joseph S.B., McKilligin E., Pei L., Watson M.A., Collins A.R., Laffitte B.A., Chen M., Noh G., Goodman J., Hagger G.N. (2002). Synthetic LXR ligand inhibits the development of atherosclerosis in mice. Proc. Natl. Acad. Sci. USA.

[53] Russo-Savage L., Schulman I.G. (2021). Liver X receptors and liver physiology. Biochim. Biophys. Acta Mol. Basis Dis..

[54] Laffitte B.A., Chao L.C., Li J., Walczak R., Hummasti S., Joseph S.B., Castrillo A., Wilpitz D.C., Mangelsdorf D.J., Collins J.L. (2003). Activation of liver X receptor improves glucose tolerance through coordinate regulation of glucose metabolism in liver and adipose tissue. Proc. Natl. Acad. Sci. USA.

[55] Zuercher W.J., Buckholz R.G., Campobasso N., Collins J.L., Galardi C.M., Gampe R.T., Hyatt S.M., Merrihew S.L., Moore J.T., Oplinger J.A. (2010). Discovery of tertiary sulfonamides as potent liver X receptor antagonists. J. Med. Chem..

[56] Wang Y., Li X., Ren S. (2020). Cholesterol Metabolites 25-Hydroxycholesterol and 25-Hydroxycholesterol 3-Sulfate Are Potent Paired Regulators: From Discovery to Clinical Usage. Metabolites.

[57] Bai Q., Zhang X., Xu L., Kakiyama G., Heuman D., Sanyal A., Pandak W.M., Yin L., Xie W., Ren S. (2012). Oxysterol sulfation by cytosolic sulfotransferase suppresses liver X receptor/sterol regulatory element binding protein-1c signaling pathway and reduces serum and hepatic lipids in mouse models of nonalcoholic fatty liver disease. Metabolism.

[58] Ma Y., Xu L., Rodriguez-Agudo D., Li X., Heuman D.M., Hylemon P.B., Pandak W.M., Ren S. (2008). 25-Hydroxycholesterol-3-sulfate regulates macrophage lipid metabolism via the LXR/SREBP-1 signaling pathway. Am. J. Physiol. Endocrinol. Metab..

[59] Rotman Y., Sanyal A.J. (2017). Current and upcoming pharmacotherapy for non-alcoholic fatty liver disease. Gut.

[60] Xu L., Bai Q., Rodriguez-Agudo D., Hylemon P.B., Heuman D.M., Pandak W.M., Ren S. (2010). Regulation of hepatocyte lipid metabolism and inflammatory response by 25-hydroxycholesterol and 25-hydroxycholesterol-3-sulfate. Lipids.

[61] Becares N., Gage M.C., Pineda-Torra I. (2017). Posttranslational Modifications of Lipid-Activated Nuclear Receptors: Focus on Metabolism. Endocrinology.

[62] Li X., Zhang S., Blander G., Tse J.G., Krieger M., Guarente L. (2007). SIRT1 deacetylates and positively regulates the nuclear receptor LXR. Mol. Cell.

[63] Defour A., Dessalle K., Castro Perez A., Poyot T., Castells J., Gallot Y.S., Durand C., Euthine V., Gu Y., Béchet D. (2012). Sirtuin 1 regulates SREBP-1c expression in a LXR-dependent manner in skeletal muscle. PLoS ONE.

[64] Hwahng S.H., Ki S.H., Bae E.J., Kim H.E., Kim S.G. (2009). Role of adenosine monophosphate-activated protein kinase-p70 ribosomal S6 kinase-1 pathway in repression of liver X receptor-alpha-dependent lipogenic gene induction and hepatic steatosis by a novel class of dithiolethiones. Hepatology.

[65] Cho K., Chung J.Y., Cho S.K., Shin H., Jang I., Park J., Yu K., Cho J. (2015). Antihyperglycemic mechanism of metformin occurs via the AMPK/LXRα/POMC pathway. Sci. Rep..

[66] Mitro N., Mak P.A., Vargas L., Godio C., Hampton E., Molteni V., Kreusch A., Saez E. (2007). The nuclear receptor LXR is a glucose sensor. Nature.

[67] Anthonisen E.H., Berven L., Holm S., Nygård M., Nebb H.I., Grønning-Wang L.M. (2010). Nuclear receptor liver X receptor is O-GlcNAc-modified in response to glucose. J. Biol. Chem..

[68] Hart G.W., Slawson C., Ramirez-Correa G., Lagerlof O. (2011). Cross talk between O-GlcNAcylation and phosphorylation: Roles in signaling, transcription, and chronic disease. Annu. Rev. Biochem..

[69] Ghisletti S., Huang W., Ogawa S., Pascual G., Lin M., Willson T.M., Rosenfeld M.G., Glass C.K. (2007). Parallel SUMOylation-dependent pathways mediate gene- and signal-specific transrepression by LXRs and PPARgamma. Mol. Cell.

[70] Turner N., Kowalski G.M., Leslie S.J., Risis S., Yang C., Lee-Young R.S., Babb J.R., Meikle P.J., Lancaster G.I., Henstridge D.C. (2013). Distinct patterns of tissue-specific lipid accumulation during the induction of insulin resistance in mice by high-fat feeding. Diabetologia.

[71] Meex R.C., Hoy A.J., Morris A., Brown R.D., Lo J.C.Y., Burke M., Goode R.J.A., Kingwell B.A., Kraakman M.J., Febbraio M.A. (2015). Fetuin B Is a Secreted Hepatocyte Factor Linking Steatosis to Impaired Glucose Metabolism. Cell Metab..

[72] Ahn S.B., Jang K., Jun D.W., Lee B.H., Shin K.J. (2014). Expression of liver X receptor correlates with intrahepatic inflammation and fibrosis in patients with nonalcoholic fatty liver disease. Dig. Dis. Sci..

[73] Samuel V.T., Shulman G.I. (2012). Mechanisms for insulin resistance: Common threads and missing links. Cell.

[74] Zhou J., Febbraio M., Wada T., Zhai Y., Kuruba R., He J., Lee J.H., Khadem S., Ren S., Li S. (2008). Hepatic fatty acid transporter Cd36 is a common target of LXR, PXR, and PPARgamma in promoting steatosis. Gastroenterology.

[75] Joseph S.B., Laffitte B.A., Patel P.H., Watson M.A., Matsukuma K.E., Walczak R., Collins J.L., Osborne T.F., Tontonoz P. (2002). Direct and indirect mechanisms for regulation of fatty acid synthase gene expression by liver X receptors. J. Biol. Chem..

[76] Schultz J.R., Tu H., Luk A., Repa J.J., Medina J.C., Li L., Schwendner S., Wang S., Thoolen M., Mangelsdorf D.J. (2000). Role of LXRs in control of lipogenesis. Genes Dev..

[77] Repa J.J., Liang G., Ou J., Bashmakov Y., Lobaccaro J.M., Shimomura I., Shan B., Brown M.S., Goldstein J.L., Mangelsdorf D.J. (2000). Regulation of mouse sterol regulatory element-binding protein-1c gene (SREBP-1c) by oxysterol receptors, LXRalpha and LXRbeta. Genes Dev..

[78] Wang B., Tontonoz P. (2018). Liver X receptors in lipid signalling and membrane homeostasis. Nat. Rev. Endocrinol..

[79] Peet D.J., Turley S.D., Ma W., Janowski B.A., Lobaccaro J.M., Hammer R.E., Mangelsdorf D.J. (1998). Cholesterol and bile acid metabolism are impaired in mice lacking the nuclear oxysterol receptor LXR alpha. Cell.

[80] Softic S., Cohen D.E., Kahn C.R. (2016). Role of Dietary Fructose and Hepatic De Novo Lipogenesis in Fatty Liver Disease. Dig. Dis. Sci..

[81] Lambert J.E., Ramos-Roman M.A., Browning J.D., Parks E.J. (2014). Increased de novo lipogenesis is a distinct characteristic of individuals with nonalcoholic fatty liver disease. Gastroenterology.

[82] Beyer T.P., Schmidt R.J., Foxworthy P., Zhang Y., Dai J., Bensch W.R., Kauffman R.F., Gao H., Ryan T.P., Jiang X. (2004). Coadministration of a liver X receptor agonist and a peroxisome proliferator activator receptor-alpha agonist in Mice: Effects of nuclear receptor interplay on high-density lipoprotein and triglyceride metabolism in vivo. J. Pharmacol. Exp. Ther..

[83] Kalaany N.Y., Gauthier K.C., Zavacki A.M., Mammen P.P.A., Kitazume T., Peterson J.A., Horton J.D., Garry D.J., Bianco A.C., Mangelsdorf D.J. (2005). LXRs regulate the balance between fat storage and oxidation. Cell Metab..

[84] Korach-André M., Archer A., Barros R.P., Parini P., Gustafsson J. (2011). Both liver-X receptor (LXR) isoforms control energy expenditure by regulating brown adipose tissue activity. Proc. Natl. Acad. Sci. USA.

[85] Kim Y.S., Nam H.J., Han C.Y., Joo M.S., Jang K., Jun D.W., Kim S.G. (2021). Liver X Receptor Alpha Activation Inhibits Autophagy and Lipophagy in Hepatocytes by Dysregulating Autophagy-Related 4B Cysteine Peptidase and Rab-8B, Reducing Mitochondrial Fuel Oxidation. Hepatology.

[86] Stenson B.M., Rydén M., Steffensen K.R., Wåhlén K., Pettersson A.T., Jocken J.W., Arner P., Laurencikiene J. (2009). Activation of liver X receptor regulates substrate oxidation in white adipocytes. Endocrinology.

[87] Janowski B.A., Willy P.J., Devi T.R., Falck J.R., Mangelsdorf D.J. (1996). An oxysterol signalling pathway mediated by the nuclear receptor LXR alpha. Nature.

[88] Lehmann J.M., Kliewer S.A., Moore L.B., Smith-Oliver T.A., Oliver B.B., Su J.L., Sundseth S.S., Winegar D.A., Blanchard D.E., Spencer T.A. (1997). Activation of the nuclear receptor LXR by oxysterols defines a new hormone response pathway. J. Biol. Chem..

[89] Alberti S., Schuster G., Parini P., Feltkamp D., Diczfalusy U., Rudling M., Angelin B., Björkhem I., Pettersson S., Gustafsson J.A. (2001). Hepatic cholesterol metabolism and resistance to dietary cholesterol in LXRbeta-deficient mice. J. Clin. Investig..

[90] Yu L., York J., von Bergmann K., Lutjohann D., Cohen J.C., Hobbs H.H. (2003). Stimulation of cholesterol excretion by the liver X receptor agonist requires ATP-binding cassette transporters G5 and G8. J. Biol. Chem..

[91] Zhang Y., Repa J.J., Gauthier K., Mangelsdorf D.J. (2001). Regulation of lipoprotein lipase by the oxysterol receptors, LXRalpha and LXRbeta. J. Biol. Chem..

[92] Yasuda T., Grillot D., Billheimer J.T., Briand F., Delerive P., Huet S., Rader D.J. (2010). Tissue-specific liver X receptor activation promotes macrophage reverse cholesterol transport in vivo. Arterioscler. Thromb. Vasc. Biol..

[93] Repa J.J., Berge K.E., Pomajzl C., Richardson J.A., Hobbs H., Mangelsdorf D.J. (2002). Regulation of ATP-binding cassette sterol transporters ABCG5 and ABCG8 by the liver X receptors alpha and beta. J. Biol. Chem..

[94] Morello F., de Boer R.A., Steffensen K.R., Gnecchi M., Chisholm J.W., Boomsma F., Anderson L.M., Lawn R.M., Gustafsson J., Lopez-Ilasaca M. (2005). Liver X receptors alpha and beta regulate renin expression in vivo. J. Clin. Investig..

[95] Tamura K., Chen Y.E., Horiuchi M., Chen Q., Daviet L., Yang Z., Lopez-Ilasaca M., Mu H., Pratt R.E., Dzau V.J. (2000). LXRalpha functions as a cAMP-responsive transcriptional regulator of gene expression. Proc. Natl. Acad. Sci. USA.

[96] Sabol S.L., Brewer H.B., Santamarina-Fojo S. (2005). The human ABCG1 gene: Identification of LXR response elements that modulate expression in macrophages and liver. J. Lipid Res..

[97] Venkateswaran A., Repa J.J., Lobaccaro J.M., Bronson A., Mangelsdorf D.J., Edwards P.A. (2000). Human white/murine ABC8 mRNA levels are highly induced in lipid-loaded macrophages. A transcriptional role for specific oxysterols. J. Biol. Chem..

[98] Hong C., Walczak R., Dhamko H., Bradley M.N., Marathe C., Boyadjian R., Salazar J.V., Tontonoz P. (2011). Constitutive activation of LXR in macrophages regulates metabolic and inflammatory gene expression: Identification of ARL7 as a direct target. J. Lipid Res..

[99] Laffitte B.A., Joseph S.B., Chen M., Castrillo A., Repa J., Wilpitz D., Mangelsdorf D., Tontonoz P. (2003). The phospholipid transfer protein gene is a liver X receptor target expressed by macrophages in atherosclerotic lesions. Mol. Cell Biol..

[100] Luo Y., Tall A.R. (2000). Sterol upregulation of human CETP expression in vitro and in transgenic mice by an LXR element. J. Clin. Investig..

[101] Scotti E., Calamai M., Goulbourne C.N., Zhang L., Hong C., Lin R.R., Choi J., Pilch P.F., Fong L.G., Zou P. (2013). IDOL stimulates clathrin-independent endocytosis and multivesicular body-mediated lysosomal degradation of the low-density lipoprotein receptor. Mol. Cell Biol..

[102] Hong C., Duit S., Jalonen P., Out R., Scheer L., Sorrentino V., Boyadjian R., Rodenburg K.W., Foley E., Korhonen L. (2010). The E3 ubiquitin ligase IDOL induces the degradation of the low density lipoprotein receptor family members VLDLR and ApoER2. J. Biol. Chem..

[103] Zelcer N., Hong C., Boyadjian R., Tontonoz P. (2009). LXR regulates cholesterol uptake through Idol-dependent ubiquitination of the LDL receptor. Science.

[104] Kirchgessner T.G., Sleph P., Ostrowski J., Lupisella J., Ryan C.S., Liu X., Fernando G., Grimm D., Shipkova P., Zhang R. (2016). Beneficial and Adverse Effects of an LXR Agonist on Human Lipid and Lipoprotein Metabolism and Circulating Neutrophils. Cell Metab..

[105] Hong C., Marshall S.M., McDaniel A.L., Graham M., Layne J.D., Cai L., Scotti E., Boyadjian R., Kim J., Chamberlain B.T. (2014). The LXR-Idol axis differentially regulates plasma LDL levels in primates and mice. Cell Metab..

[106] Wong J., Quinn C.M., Brown A.J. (2006). SREBP-2 positively regulates transcription of the cholesterol efflux gene, ABCA1, by generating oxysterol ligands for LXR. Biochem. J..

[107] Sallam T., Jones M.C., Gilliland T., Zhang L., Wu X., Eskin A., Sandhu J., Casero D., Vallim T.Q.d.A., Hong C. (2016). Feedback modulation of cholesterol metabolism by the lipid-responsive non-coding RNA LeXis. Nature.

[108] Zhang L., Rajbhandari P., Priest C., Sandhu J., Wu X., Temel R., Castrillo A., de Aguiar Vallim T.Q., Sallam T., Tontonoz P. (2017). Inhibition of cholesterol biosynthesis through RNF145-dependent ubiquitination of SCAP. eLife.

[109] Joseph S.B., Castrillo A., Laffitte B.A., Mangelsdorf D.J., Tontonoz P. (2003). Reciprocal regulation of inflammation and lipid metabolism by liver X receptors. Nat. Med..

[110] Ogawa D., Stone J.F., Takata Y., Blaschke F., Chu V.H., Towler D.A., Law R.E., Hsueh W.A., Bruemmer D. (2005). Liver x receptor agonists inhibit cytokine-induced osteopontin expression in macrophages through interference with activator protein-1 signaling pathways. Circ. Res..

[111] Terasaka N., Hiroshima A., Ariga A., Honzumi S., Koieyama T., Inaba T., Fujiwara T. (2005). Liver X receptor agonists inhibit tissue factor expression in macrophages. FEBS J..

[112] Blaschke F., Takata Y., Caglayan E., Collins A., Tontonoz P., Hsueh W.A., Tangirala R.K. (2006). A nuclear receptor corepressor-dependent pathway mediates suppression of cytokine-induced C-reactive protein gene expression by liver X receptor. Circ. Res..

[113] Venteclef N., Jakobsson T., Ehrlund A., Damdimopoulos A., Mikkonen L., Ellis E., Nilsson L., Parini P., Jänne O.A., Gustafsson J. (2010). GPS2-dependent corepressor/SUMO pathways govern anti-inflammatory actions of LRH-1 and LXRbeta in the hepatic acute phase response. Genes Dev..

[114] Kiss E., Popovic Z., Bedke J., Wang S., Bonrouhi M., Gretz N., Stettner P., Teupser D., Thiery J., Porubsky S. (2011). Suppression of chronic damage in renal allografts by Liver X receptor (LXR) activation relevant contribution of macrophage LXRα. Am. J. Pathol..

[115] Castrillo A., Joseph S.B., Vaidya S.A., Haberland M., Fogelman A.M., Cheng G., Tontonoz P. (2003). Crosstalk between LXR and toll-like receptor signaling mediates bacterial and viral antagonism of cholesterol metabolism. Mol. Cell.

[116] Thomas D.G., Doran A.C., Fotakis P., Westerterp M., Antonson P., Jiang H., Jiang X., Gustafsson J., Tabas I., Tall A.R. (2018). LXR Suppresses Inflammatory Gene Expression and Neutrophil Migration through cis-Repression and Cholesterol Efflux. Cell Rep..

[117] Ito A., Hong C., Rong X., Zhu X., Tarling E.J., Hedde P.N., Gratton E., Parks J., Tontonoz P. (2015). LXRs link metabolism to inflammation through Abca1-dependent regulation of membrane composition and TLR signaling. eLife.

[118] Valledor A.F., Hsu L., Ogawa S., Sawka-Verhelle D., Karin M., Glass C.K. (2004). Activation of liver X receptors and retinoid X receptors prevents bacterial-induced macrophage apoptosis. Proc. Natl. Acad. Sci. USA.

[119] Joseph S.B., Bradley M.N., Castrillo A., Bruhn K.W., Mak P.A., Pei L., Hogenesch J., O’connell R.M., Cheng G., Saez E. (2004). LXR-dependent gene expression is important for macrophage survival and the innate immune response. Cell.

[120] Korf H., Vander Beken S., Romano M., Steffensen K.R., Stijlemans B., Gustafsson J., Grooten J., Huygen K. (2009). Liver X receptors contribute to the protective immune response against Mycobacterium tuberculosis in mice. J. Clin. Investig..

[121] Smoak K., Madenspacher J., Jeyaseelan S., Williams B., Dixon D., Poch K.R., Nick J.A., Worthen G.S., Fessler M.B. (2008). Effects of liver X receptor agonist treatment on pulmonary inflammation and host defense. J. Immunol..

[122] Birrell M.A., Catley M.C., Hardaker E., Wong S., Willson T.M., McCluskie K., Leonard T., Farrow S.N., Collins J.L., Haj-Yahia S. (2007). Novel role for the liver X nuclear receptor in the suppression of lung inflammatory responses. J. Biol. Chem..

[123] Wang Y.Y., Dahle M.K., Steffensen K.R., Reinholt F.P., Collins J.L., Thiemermann C., Aasen A.O., Gustafsson J., Wang J.E. (2009). Liver X receptor agonist GW3965 dose-dependently regulates lps-mediated liver injury and modulates posttranscriptional TNF-alpha production and p38 mitogen-activated protein kinase activation in liver macrophages. Shock.

[124] Kim Y.M., Kim T.H., Kim Y.W., Yang Y.M., Ryu D.H., Hwang S.J., Lee J.R., Kim S.C., Kim S.G. (2010). Inhibition of liver X receptor-α-dependent hepatic steatosis by isoliquiritigenin, a licorice antioxidant flavonoid, as mediated by JNK1 inhibition. Free. Radic. Biol. Med..

[125] Listenberger L.L., Han X., Lewis S.E., Cases S., Farese R.V., Ory D.S., Schaffer J.E. (2003). Triglyceride accumulation protects against fatty acid-induced lipotoxicity. Proc. Natl. Acad. Sci. USA.

[126] Teratani T., Tomita K., Suzuki T., Oshikawa T., Yokoyama H., Shimamura K., Tominaga S., Hiroi S., Irie R., Okada Y. (2012). A high-cholesterol diet exacerbates liver fibrosis in mice via accumulation of free cholesterol in hepatic stellate cells. Gastroenterology.

[127] Tomita K., Teratani T., Suzuki T., Shimizu M., Sato H., Narimatsu K., Okada Y., Kurihara C., Irie R., Yokoyama H. (2014). Free cholesterol accumulation in hepatic stellate cells: Mechanism of liver fibrosis aggravation in nonalcoholic steatohepatitis in mice. Hepatology.

[128] Beaven S.W., Wroblewski K., Wang J., Hong C., Bensinger S., Tsukamoto H., Tontonoz P. (2011). Liver X receptor signaling is a determinant of stellate cell activation and susceptibility to fibrotic liver disease. Gastroenterology.

[129] Wang X., Cai B., Yang X., Sonubi O.O., Zheng Z., Ramakrishnan R., Shi H., Valenti L., Pajvani U.B., Sandhu J. (2020). Cholesterol Stabilizes TAZ in Hepatocytes to Promote Experimental Non-alcoholic Steatohepatitis. Cell Metab..

[130] Becares N., Gage M.C., Voisin M., Shrestha E., Martin-Gutierrez L., Liang N., Louie R., Pourcet B., Pello O.M., Luong T.V. (2019). Impaired LXRα Phosphorylation Attenuates Progression of Fatty Liver Disease. Cell Rep..

[131] Raselli T., Hearn T., Wyss A., Atrott K., Peter A., Frey-Wagner I., Spalinger M.R., Maggio E.M., Sailer A.W., Schmitt J. (2019). Elevated oxysterol levels in human and mouse livers reflect nonalcoholic steatohepatitis. J. Lipid Res..

[132] Ikegami T., Hyogo H., Honda A., Miyazaki T., Tokushige K., Hashimoto E., Inui K., Matsuzaki Y., Tazuma S. (2012). Increased serum liver X receptor ligand oxysterols in patients with non-alcoholic fatty liver disease. J. Gastroenterol..

[133] Moldavski O., Zushin P.H., Berdan C.A., Van Eijkeren R.J., Jiang X., Qian M., Ory D.S., Covey D.F., Nomura D.K., Stahl A. (2021). 4β-Hydroxycholesterol is a prolipogenic factor that promotes SREBP1c expression and activity through the liver X receptor. J. Lipid Res..

[134] Li D., Long W., Huang R., Chen Y., Xia M. (2018). 27-Hydroxycholesterol Inhibits Sterol Regulatory Element-Binding Protein 1 Activation and Hepatic Lipid Accumulation in Mice. Obesity.

[135] Adams C.M., Reitz J., De Brabander J.K., Feramisco J.D., Li L., Brown M.S., Goldstein J.L. (2004). Cholesterol and 25-hydroxycholesterol inhibit activation of SREBPs by different mechanisms, both involving SCAP and Insigs. J. Biol. Chem..

[136] Wang Y., Chen L., Pandak W.M., Heuman D., Hylemon P.B., Ren S. (2020). High Glucose Induces Lipid Accumulation via 25-Hydroxycholesterol DNA-CpG Methylation. iScience.

[137] Zhang Y., Breevoort S.R., Angdisen J., Fu M., Schmidt D.R., Holmstrom S.R., Kliewer S.A., Mangelsdorf D.J., Schulman I.G. (2012). Liver LXRα expression is crucial for whole body cholesterol homeostasis and reverse cholesterol transport in mice. J. Clin. Investig..

[138] Hu B., Unwalla R.J., Goljer I., Jetter J.W., Quinet E.M., Berrodin T.J., Basso M.D., Feingold I.B., Nilsson A.G., Wilhelmsson A. (2010). Identification of phenylsulfone-substituted quinoxaline (WYE-672) as a tissue selective liver X-receptor (LXR) agonist. J. Med. Chem..

[139] Ratni H., Blum-Kaelin D., Dehmlow H., Hartman P., Jablonski P., Masciadri R., Maugeais C., Patiny-Adam A., Panday N., Wright M. (2009). Discovery of tetrahydro-cyclopenta[b]indole as selective LXRs modulator. Bioorganic Med. Chem. Lett..

[140] Peng D., Hiipakka R.A., Dai Q., Guo J., Reardon C.A., Getz G.S., Liao S. (2008). Antiatherosclerotic effects of a novel synthetic tissue-selective steroidal liver X receptor agonist in low-density lipoprotein receptor-deficient mice. J. Pharmacol. Exp. Ther..

[141] Tice C.M., Noto P.B., Fan K.Y., Zhuang L., Lala D.S., Singh S.B. (2014). The medicinal chemistry of liver X receptor (LXR) modulators. J. Med. Chem..

[142] Muse E.D., Yu S., Edillor C.R., Tao J., Spann N.J., Troutman T.D., Seidman J.S., Henke A., Roland J.T., Ozeki K.A. (2018). Cell-specific discrimination of desmosterol and desmosterol mimetics confers selective regulation of LXR and SREBP in macrophages. Proc. Natl. Acad. Sci. USA.

[143] Katz A., Udata C., Ott E., Hickey L., Burczynski M.E., Burghart P., Vesterqvist O., Meng X. (2009). Safety, pharmacokinetics, and pharmacodynamics of single doses of LXR-623, a novel liver X-receptor agonist, in healthy participants. J. Clin. Pharmacol..

[144] Quinet E.M., Basso M.D., Halpern A.R., Yates D.W., Steffan R.J., Clerin V., Resmini C., Keith J.C., Berrodin T.J., Feingold I. (2009). LXR ligand lowers LDL cholesterol in primates, is lipid neutral in hamster, and reduces atherosclerosis in mouse. J. Lipid Res..

[145] (2011). Placebo-Controlled, Ascending, Multiple-Dose Study to Evaluate the Safety, Pharmacokinetics and Pharmacodynamics of BMS-779788 in Healthy Subjects. https://clinicaltrials.gov/ct2/show/NCT00836602.

[146] (2018). A Randomized, Placebo-Controlled, Multiple Ascending Dose Study To Assess The Safety, Pharmacokinetics and Pharmacodynamics Of CS-8080 In Healthy Volunteers. https://clinicaltrials.gov/ct2/show/NCT00796575.

[147] Fessler M.B. (2018). The Challenges and Promise of Targeting the Liver X Receptors for Treatment of Inflammatory Disease. Pharmacol. Ther..

